# 14-3-3σ Regulates β-Catenin-Mediated Mouse Embryonic Stem Cell Proliferation by Sequestering GSK-3β

**DOI:** 10.1371/journal.pone.0040193

**Published:** 2012-06-29

**Authors:** Tzu-Ching Chang, Chia-Chia Liu, En-Wei Hsing, Shu-Man Liang, Ya-Hui Chi, Li-Ying Sung, Shau-Ping Lin, Tang-Long Shen, Bor-Sheng Ko, B. Linju Yen, Shaw-Fang Yet, Kenneth K. Wu, Jun-Yang Liou

**Affiliations:** 1 Institute of Cellular and System Medicine, National Health Research Institutes, Zhunan, Taiwan; 2 Institute of Biotechnology, National Taiwan University, Taipei, Taiwan; 3 Department of Plant Pathology and Microbiology, National Taiwan University, Taipei, Taiwan; 4 Department of Internal Medicine, National Taiwan University Hospital, Taipei, Taiwan; William Harvey Research Institute, Barts and The London School of Medicine and Dentistry, Queen Mary University of London, United Kingdom

## Abstract

**Background:**

Pluripotent embryonic stem cells are considered to be an unlimited cell source for tissue regeneration and cell-based therapy. Investigating the molecular mechanism underlying the regulation of embryonic stem cell expansion is thus important. 14-3-3 proteins are implicated in controlling cell division, signaling transduction and survival by interacting with various regulatory proteins. However, the function of 14-3-3 in embryonic stem cell proliferation remains unclear.

**Methodology and Principal Findings:**

In this study, we show that all seven 14-3-3 isoforms were detected in mouse embryonic stem cells. Retinoid acid suppressed selectively the expression of 14-3-3σ isoform. Knockdown of 14-3-3σ with siRNA reduced embryonic stem cell proliferation, while only 14-3-3σ transfection increased cell growth and partially rescued retinoid acid-induced growth arrest. Since the growth-enhancing action of 14-3-3σ was abrogated by β-catenin knockdown, we investigated the influence of 14-3-3σ overexpression on β-catenin/GSK-3β. 14-3-3σ bound GSK-3β and increased GSK-3β phosphorylation in a PI-3K/Akt-dependent manner. It disrupted β-catenin binding by the multiprotein destruction complex. 14-3-3σ overexpression attenuated β-catenin phosphorylation and rescued the decline of β-catenin induced by retinoid acid. Furthermore, 14-3-3σ enhanced Wnt3a-induced β-catenin level and GSK-3β phosphorylation. DKK, an inhibitor of Wnt signaling, abolished Wnt3a-induced effect but did not interfere GSK-3β/14-3-3σ binding.

**Significance:**

Our findings show for the first time that 14-3-3σ plays an important role in regulating mouse embryonic stem cell proliferation by binding and sequestering phosphorylated GSK-3β and enhancing Wnt-signaled GSK-3β inactivation. 14-3-3σ is a novel target for embryonic stem cell expansion.

## Introduction

Embryonic stem (ES) cells are pluripotent cells that possess self-renewal properties and retain the capacity for differentiation into all 3 germ layer cells [1], [2]. Because of their high proliferation capability, pluripotency and low immunogenicity, ES cells are considered to be a valuable source for cell therapy, tissue regeneration, drug testing and developmental biology [3], [4]. ES cell proliferation and renewal are maintained by diverse factors that activate the renewal genetic program via selective signaling pathways [5], [6] among which the β-catenin pathway plays a pivotal role [7], [8]. At the basal state, β-catenin is associated with a multiprotein destruction complex composed of APC (adenomatous polyposis coli), axin, casein kinase 2 and glycogen synthase kinase 3β (GSK-3β) where it is phosphorylated and degraded via ubiquitin/proteasome [9]–[11]. Upon Wnt activation through binding to frizzled and/or LRP5/6 receptors, disheveled (Dvl) displaces GSK-3β from the APC complex resulting in reduced β-catenin degradation, and increased cytosolic β-catenin which is translocated to nucleus where it is associated with Tcf/Lef transcription factor to drive the expression of renewal and proliferative genes. Experimental data have provided convincing evidence for the crucial role of GSK-3β/β-catenin in ES cell renewal [12]–[14]. GSK-3β is a serine/threonine protein kinase which was originally discovered as an enzyme that phosphorylates and inactivates glycogen synthase in response to insulin, and was subsequently reported to phosphorylate β-catenin and facilitate β-catenin ubiquitination and degradation [15]. GSK-3β inhibition was shown to maintain ES cells in the renewal state [14]. Thus, GSK-3β occupies a central position in controlling β-catenin and ES cell renewal and differentiation. Its activity must be tightly regulated. However, little is known about its regulation in ES cells. We propose in this study that 14-3-3 proteins regulate GSK-3β availability.

14-3-3 proteins are 28- to 33-kDa acidic polypeptides found in all eukaryotic organisms [16]–[18]. 7 members (β, γ, ε, η, σ, θ/τ and ζ) are found in mammals. These isoforms form homo- or hetero-dimers to serve as scaffolds. At least 200 proteins are reported to interact with 14-3-3 [16]. Through binding to various classes of proteins including enzymes, transcription factors, cytoskeletal proteins, signaling molecules, apoptosis factors and tumor suppressors, 14-3-3 proteins are involved in diverse cellular functions and pathophysiological processes [17]. 14-3-3 isoforms have been reported to regulate GSK-3β. 14-3-3ζ was reported to bind GSK-3β, and stimulates tau phosphorylation in the brain [19]. 14-3-3β interacts with Ser9-phosphorylated GSK-3β to control neuronal survival [20]. 14-3-3 was also reported to interact with β-catenin and modify its transcriptional activity. 14-3-3ζ interacts with β-catenin and enhances β-catenin transactivation action [21]. On the other hand, 14-3-3ζ was reported to interact with Chibby protein to export β-catenin from nucleus and consequently attenuate the β-catenin transcriptional activity [22]. These results indicate that 14-3-3 proteins are functionally complex. Little is known about 14-3-3 proteins in ES cells, let alone their roles in ES cell renewal and proliferation. In this study, we investigated the involvement of 14-3-3 proteins in regulating mouse ES cell (mESC) proliferation. We provide evidence that 14-3-3σ isoform regulates mESC proliferation by binding and sequestering GSK-3β and enhancing Wnt3a-induced GSK-3β phosphorylation and inactivation. 14-3-3σ overexpression rescues retinoid acid (RA)-induced growth arrest by increasing GSK-3β phosphorylation and β-catenin level.

**Figure 1:**
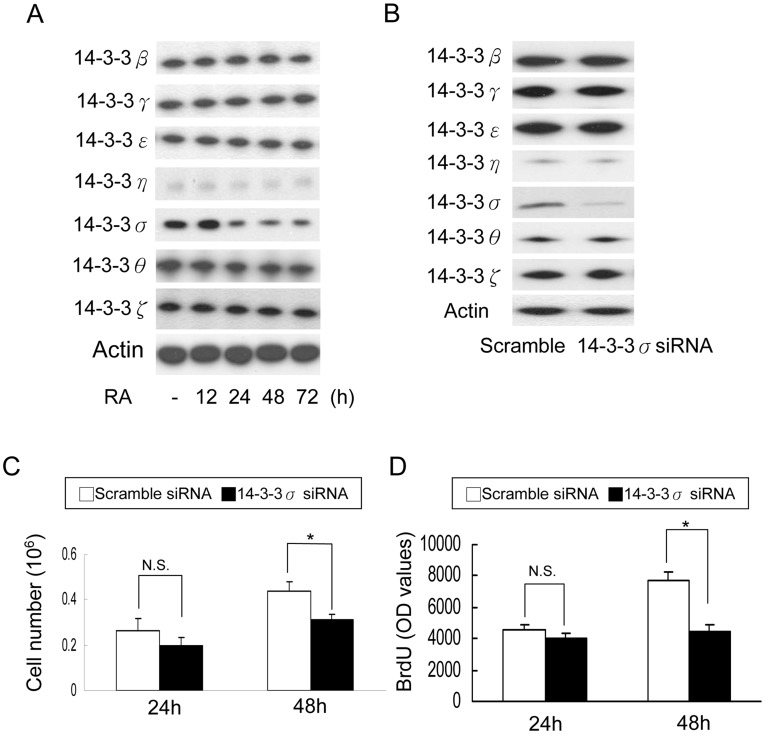
14-3-3σ is involved in mESC proliferation. (A) CCE cells were treated with 10 µg/mL retinoic acid (RA) for 12 to 72 h as indicated, and protein levels of 14-3-3 isoforms were determined by Western blot analysis. Actin was used as loading control. (B) CCE cells were transfected with scramble or 14-3-3σ siRNA and each 14-3-3σ isoform protein was determined by Western blot analysis after 48 h transfection. (C) Viable CCE cells were determined by trypan blue assay, and (D) BrdU incorporation was analyzed. Error bars are mean ± s.d. (n = 3). N.S. denotes statistically not significant; *, *p*<0.05.

**Figure 2:**
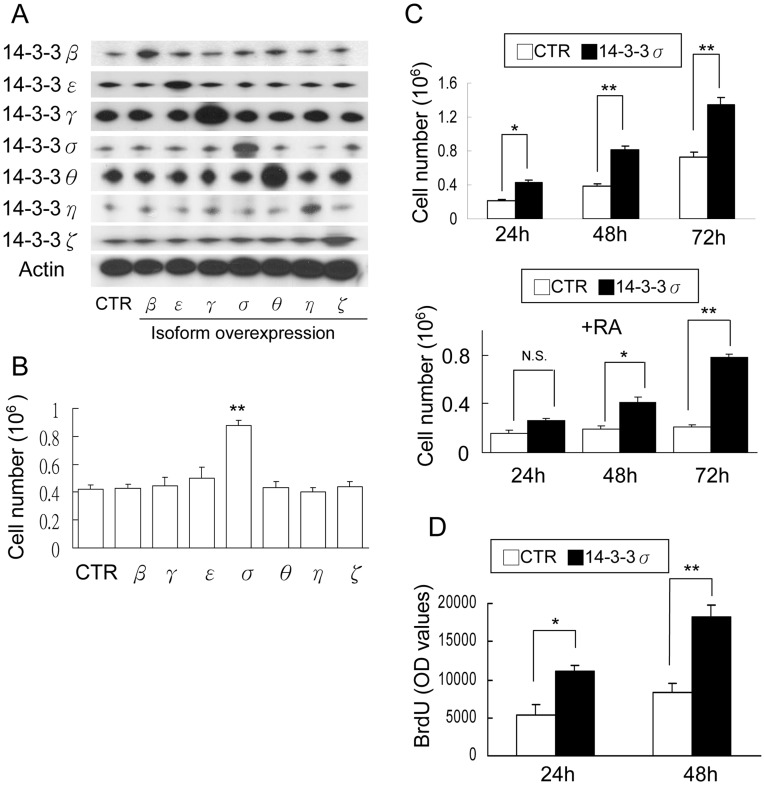
14-3-3σ overexpression increases CCE proliferation. (A) and (B) CCE cells were transfected with control (CTR) or each 14-3-3 isoform and (A) all isoform proteins were analyzed by Western blotting, (B) cell numbers were counted. (C) CCE cells transfected with CTR or 14-3-3σ vectors were treated with (lower panel) or without RA (upper panel) and cell number were counted. (D) CCE cells transfected with CTR or 14-3-3σ vectors and BrdU incorporation were determined. Error bars are mean ± s.d. (n = 3). N.S. denotes statistically not significant; *p<0.05; **p<0.01.

## Materials and Methods

### Cell Culture and Reagents

CCE, a mESC line derived from the 129/Sv mouse strain, was obtained from StemCell Technologies with permission from Drs. Robertson and Keller (Vancouver, Canada). CCE cells were cultured on gelatin-coated dishes in Dulbecco’s modified Eagle’s medium (DMEM) supplemented with 15% fetal bovine serum (Hyclone, Logan, UT, USA), 100 U/ml penicillin, 100 µg/ml streptomycin, 1 mM sodium pyruvate, 0.1 mM non-essential amino acids, and 10 ng/mL leukemia inhibitory factor at 37°C in a humidified 5% CO_2_ atmosphere [23], [24]. D3 and R1 mouse ES cells [25], [26] were cultured and maintained on feeder cells comprising mitotically inactivated primary mouse embryonic fibroblasts (MEFs) in the same medium of CCE cells. Mouse recombinant Wnt3a and Wnt inhibitor, DKK-1 were from Calbiochem (San Diego, CA, USA). The PI3-K inhibitor wortmannin was from Sigma (St. Louis, MO, USA).

### Plasmid Constructs and Transfection

cDNA of each 14-3-3 isoform (β, γ, ε, η, σ, θ and ζ) was amplified by PCR and cloned into the p3XFlag-CMV expression vector (Sigma) with the restriction enzymes HindIII and BamH1. The expression vector of β-catenin was constructed as described [27]. Small interfering RNA (siRNA) of 14-3-3σ and β-catenin was from Santa Cruz Biotechnology (Santa Cruz, CA). 14-3-3σ siRNA used for knockdown experiments comprised three RNA sequences including sc-29591A (sense: GAAGACAUGGCAGCUUUCATT, antisense: UGAAAGCUGCCAUGUCUUCTT); sc-29591B (sense: CCCAAACCCUGAAUGUUCATT, antisense: UGAACAUUCAGGGUUUGGGTT) and sc-29591C (sense: GUGUGACCAUGGUACCAAUTT, antisense: AUUGGUAC CAUGGUCACACTT) [28], [29]. The siRNA specific to β-catenin was sc-29209 (sense: AGCUGAUAUUGAUGGACAGTT and antisense: CUGUCCAUCAAUAUCAGCUTT) [30], [31]. Expression vectors containing GSK-3β wild-type (WT) and GSK-3β Ser9A mutant cDNA were from Addgene (plasmid 16260 and 16261) [32]. The GSK-3β Thr309A mutant cDNA plasmid was cloned using a site-directed mutagenesis kit (Stratagene, La Jolla, USA). Expression vectors and siRNA were co-transfected into mESCs using Effectene transfection reagent (Qiagen, GmbH, Hilden, Germany) as described [33], [34]. In brief, DNA plasmids or siRNA were mixed with Enhancer and Effectene at a ratio of 1 (µg) to 1.6 (µL) to 4 (µL). The adherent CCE colonies were trypsinized to yield single-cell suppression. The D3 or R1 cells on MEFs were trypsinized and pre-precipitated for 1 h to remove feeder cells. DNA (or siRNA)-Effectene mixture was added to CCE, D3 or R1 cell suspension, which was seeded on gelatin-coating plates or MEF feeder cells for 24–48 h. Cells were harvested and assayed.

**Figure 3:**
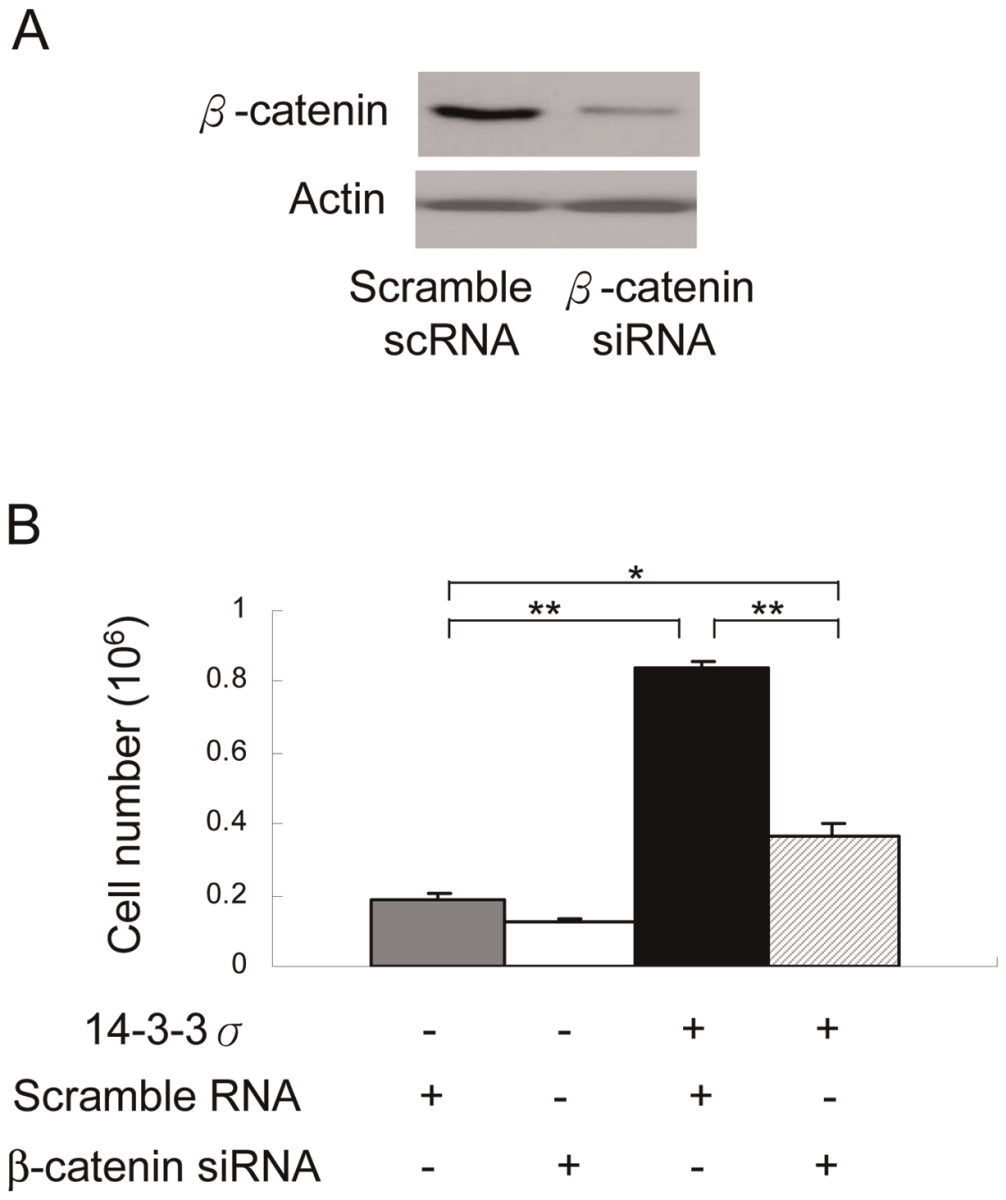
14-3-3σ-enhanced CCE proliferation is suppressed by β-catenin siRNA. (A) CCE cells were transfected with scramble or the reported β-catenin siRNA sequence and co-transfected with 14-3-3σ vectors for 48 h. β-catenin proteins were determined by Western blot analysis. (B) Viable cells were determined by trypan blue assay. Error bar is mean ± s.d. (n = 3). *p<0.05; **p<0.01.

**Figure 4:**
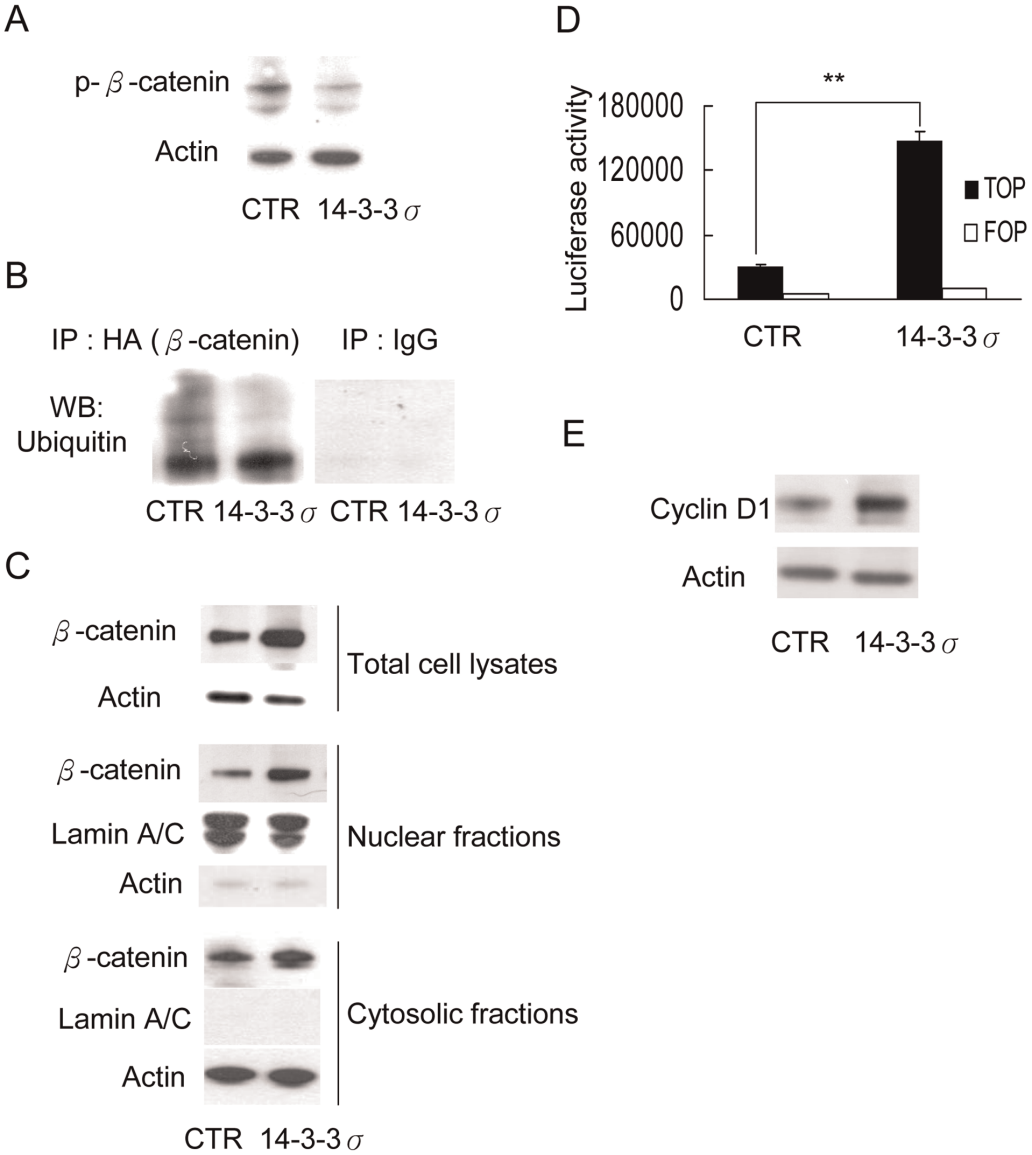
14-3-3σ overexpression enhances β-catenin stability and nuclear translocation. (A) CCE cells were transfected with control (CTR) or 14-3-3σ vectors for 48 h. Phosphorylated β-catenin (p-β-catenin) was determined by Western blot analysis. Actin was used as loading control. (B) β-catenin ubiquitination was analyzed by immunoprecipitation (IP) followed by Western blotting (WB). (C) Cell lysate and nuclear fraction were prepared from CTR and 14-3-3σ-transfected CCE cells and β-catenin was determined by Western blot analysis. LaminA/C and Actin were used as the loading control of nuclear and cytosolic fractions. (D) β-catenin transactivation activity was determined by luciferase-based TOP reporter assay. FOP reporter activity was used as a control. Data represent mean ± s.d. (n = 3). **, p<0.01. (E) Cyclin D1 expression in CTR or 14-3-3σ transfected CCE was determined by Western blot analysis. Actin was used as loading control.

### Western Blot Analysis

Western blot analysis was performed as described [35]. Briefly, cells were washed with phosphate-buffered saline (PBS) and lysed in ice-cold RIPA lysis buffer (Upstate, Lake Placid, NY) containing a protease inhibitor cocktail (Roche Diagnostics GmbH, Mannheim, Germany). The lysate was centrifuged, the supernatant collected, and protein concentration determined by the Bio-Rad Protein Assay kit (Bio-Rad Laboratories, Hercules, CA). 30 µg of supernatant proteins was applied to each lane of an SDS polyacrylamide gel. Proteins were resolved by electrophoresis and transferred to PVDF membrane (Millipore, Bedford, MA). Membranes were blocked with 5% milk, incubated with primary antibodies overnight at 4°C, washed and incubated with horseradish peroxidase-conjugated secondary antibodies for 1 hr at room temperature. The protein bands were visualized by enhanced chemiluminescence (PerkinElmer, Shelton, CT). Rabbit polyclonal antibodies against β-catenin, phosphor-β-catenin, GSK-3β, phospho-GSK-3β (Ser9), Akt, phospho-Akt, APC, axin, ubiquitin and cyclin D1 were from Cell Signaling. Mouse monoclonal or rabbit polyclonal antibodies against 14-3-3 isoforms, Oct3/4 and lamin A/C were from Santa Cruz Biotechnology. Antibodies specifically for 14-3-3 isoforms were raised against peptide regions or sequences as follows: C-terimal region of 14-3-3β, amino acids 130–170 of 14-3-3ε, 206–246 of 14-3-3γ, 205–245 of 14-3-3θ, 172–202 of 14-3-3η, 109–149 of 14-3-3ζ and the entire recombinant protein of 14-3-3σ. Mouse monoclonal antibodies against Flag, HA and actin were from Sigma.

**Figure 5:**
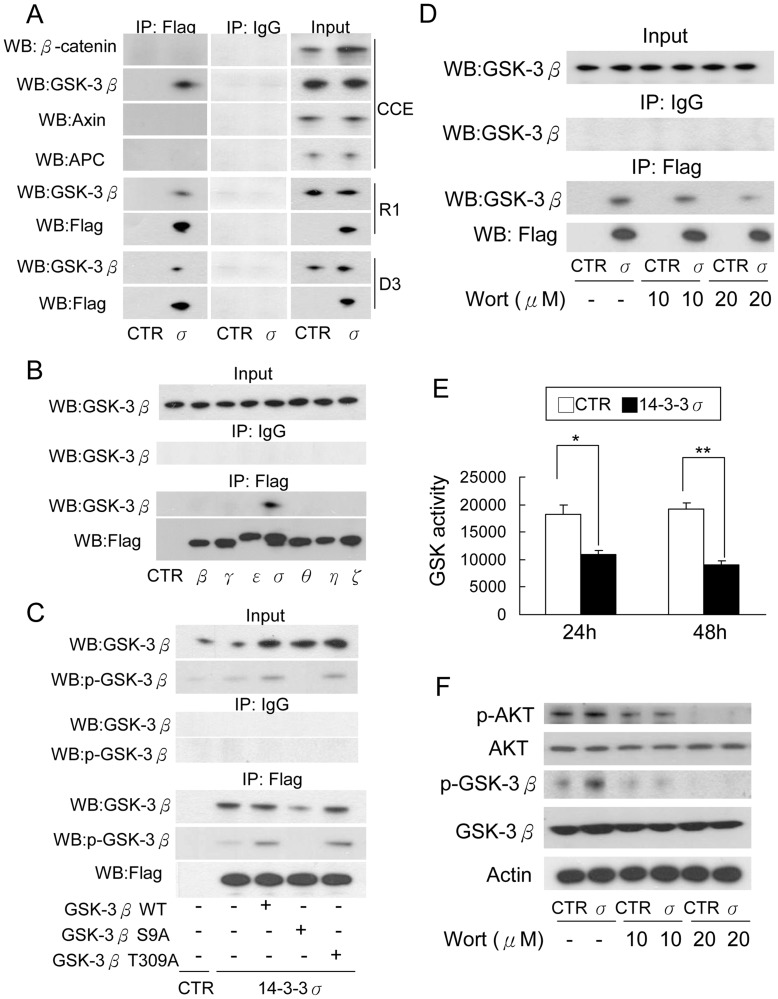
14-3-3σ interacts with GSK-3β. (A) CCE, R1 and D3 cells transfected with control or 14-3-3σ vectors were lysed and 14-3-3σ complex was pulled down by immunoprecipitation (IP) with a Flag antibody. Proteins in the complex were analyzed by Western blotting (WB). (B) CCE cells were transfected with each isoform vector. 14-3-3 complex was pulled down and GSK-3β was determined by Western blotting. (C) Association of 14-3-3σ with WT or mutant GSK-3β was determined by immunoprecipitation (IP) with a Flag Antibody followed by Western blotting (WB) with GSK-3β and p-GSK-3β antibodies. (D) CCE cells transfected with Flag-tagged 14-3-3σ (σ) or control (CTR) vectors were treated with wortmannin. 14-3-3σ complex was immunoprecipitated with a Flag antibody and GSK-3β was analyzed by Western blotting. (E) 14-3-3σ overexpression reduced GSK-3β activity. Data represents mean ± s.d. (n = 3). *, p<0.05; **, p<0.01. (F) CCE cells were transfected with control or 14-3-3σ vectors for 42 h followed by treatment with wortmannin (Wort) for 6 h. Total and phosphorylated protein levels of GSK-3β and Akt were detected by Western blot analysis.

### Preparation of Nuclear Proteins

Nuclear proteins were extracted by using an extraction kit (Chemicon). Briefly, cells were harvested and lysed with cytoplasmic buffer containing protease inhibitors for 15 min at 4°C, mixed, and centrifuged at 8,000 g for 20 min at 4°C. Supernatants were collected (cytoplasmic extraction) and pellets were resuspended in nucleus buffer containing protease inhibitors for 15 min at 4°C. The resuspended sample was mixed and centrifuged at 16,000 g for 10 min at 4°C. The supernatant containing nuclear extraction proteins was collected and stored at −80°C.

**Figure 6:**
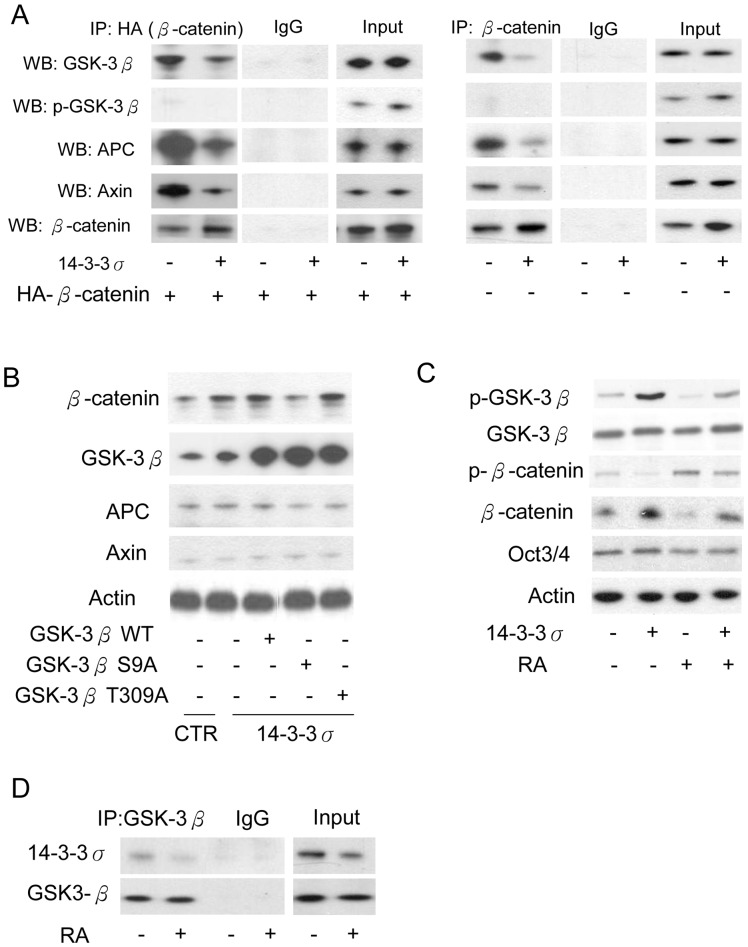
14-3-3σ reduces β-catenin association with the APC/axin/GSK-3β complex and increases β-catenin. (A) Association of HA-tagged (left panels) and endogenous (right panels) β-catenin with the APC complex was determined by IP with HA antibodies and Western blotting (WB) with the indicated antibodies. (B) CCE cells were transfected with GSK-3β WT or the indicated mutants and β-catenin, GSK-3β, APC and Axin proteins were analyzed by Western blotting. (C) CCE cells with or without 14-3-3σ overexpression were treated with RA. The indicated GSK-3β, β-catenin and Oct3/4 proteins were analyzed by Western blotting. (D) Cells were treated with or without RA for 48 h, the association of endogenous 14-3-3σ with GSK-3β was determined by IP with GSK-3β antibody and Western blotting of 14-3-3σ.

### Cell Proliferation Analysis

For cell proliferation analysis in this study, 1.5×10^5^ transfected cells were seeded (defined as 0 h) and incubated for the indicated time. Cell number was determined by trypan blue assay. Cells were trypsinized, resuspended in medium, and viable cells were counted by using a hemocytometer. Cell proliferation was analyzed with a bromodeoxyuridine (BrdU) cell proliferation assay kit (Chemicon). Briefly, BrdU, a thymidine analog, is incorporated into newly synthesized DNA as cells enter the S phase. Following partial denaturation of double-stranded DNA, BrdU was detected immunochemically with a specific mouse monoclonal antibody. The amount of BrdU was determined after the addition of IgG-peroxidase conjugated secondary antibody, peroxidase substrate and stop solution.

**Figure 7:**
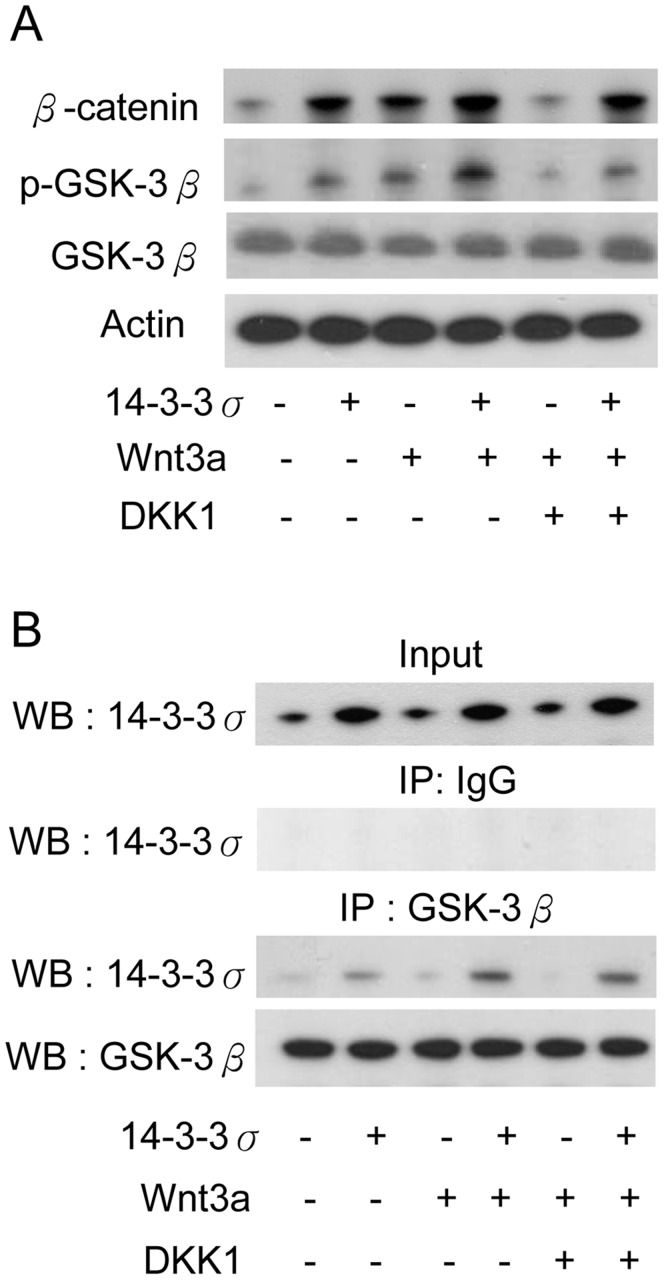
14-3-3σ enhances Wnt/β-catenin signaling. (A) CCE cells were transfected with control or 14-3-3σ vectors for 30 h followed by treatment with recombinant mouse Wnt3a (10 ng/mL) for 18 h. Recombinant mouse DKK-1 (1 ng/mL) was added 2 h before Wnt3a. Protein levels of β-catenin, GSK-3β and phosphorylated GSK-3β were detected by Western blot analysis. (B) 14-3-3σ/GSK-3β complex was determined by IP with a GSK-3β antibody, and Flag and GSK-3β proteins were analyzed by Western blotting.

### Promoter Activity Assay

β-catenin promoter activity was measured by using TOPFLASH/FOPFLASH reporter (Millipore). TOPFLASH/FOPFLASH constructs and 14-3-3σ or control vectors were incubated with Effectene transfection reagent in a 12-well plate for 48 h. Cells were washed with PBS and lysed in lysis buffer (Promega). Luciferase activity was measured with Luciferase Assay Reagent (Promega), and the emitted light was determined in a luminometer.

**Figure 8:**
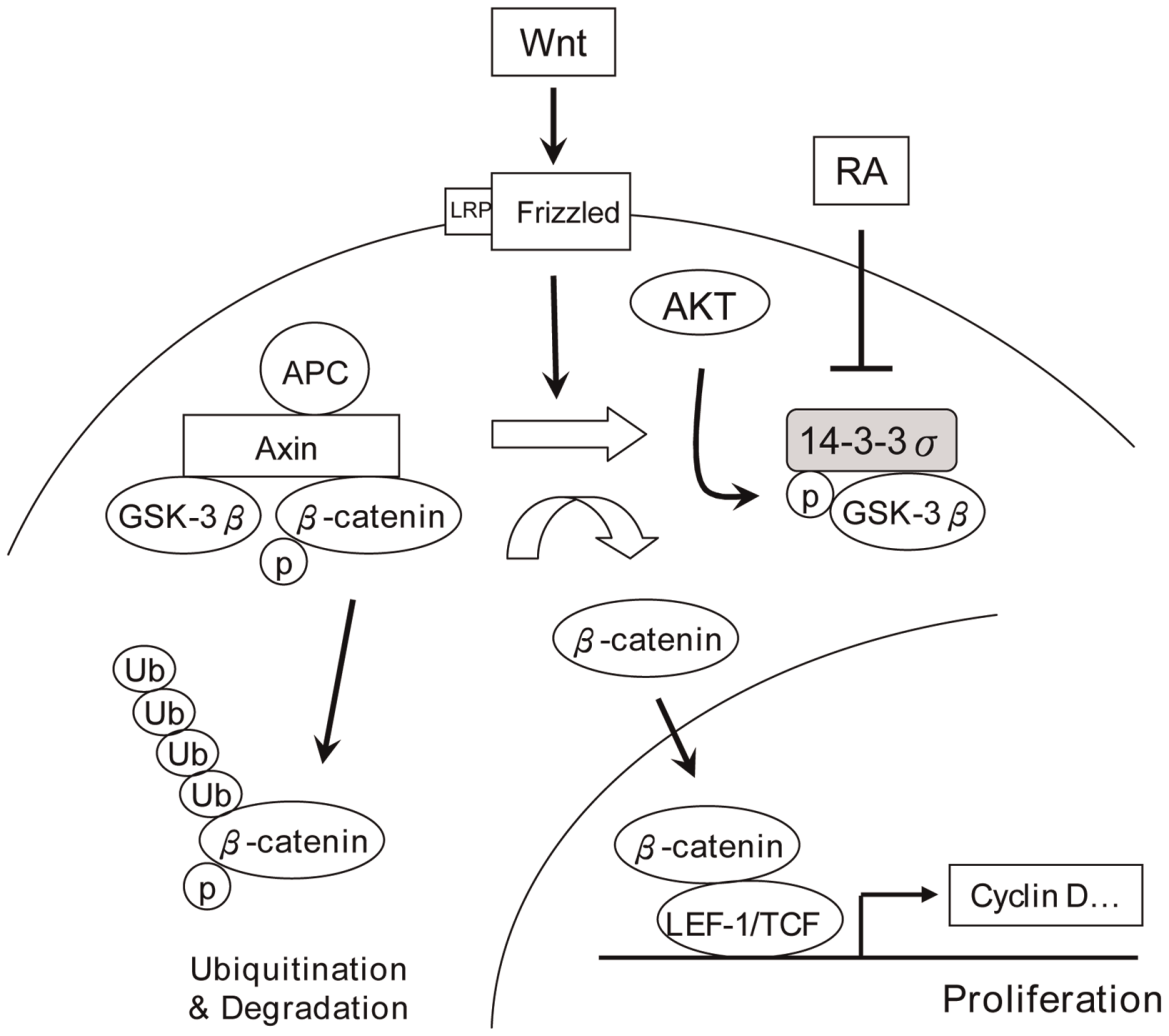
A schematic model illustrating the role of 14-3-3σ in regulating ES cell proliferation via GSK-3β/β-catenin pathway.

### Immunoprecipitation and Ubiquitination Assay

CCE cells were transfected with 14-3-3σ-Flag or co-transfected with β-catenin and/or GSK-3β for 48 h. Cells were harvested and lysed in RIPA buffer for 30 min at 4°C. After centrifugation, cell lysates were immunoprecipitated with a mouse monoclonal anti-Flag, anti-HA antibodies or mouse IgG as a control. The immunoprecipitates were resuspended in Laemmli sample buffer with 2-mercaptoethanol and boiled for 15 min. Proteins in the immunoprecipitate were separated by SDS–PAGE and analyzed by immunoblotting with rabbit polyclonal antibodies against β-catenin, GSK-3β, axin or APC. To evaluate the isoform-specific interaction of 14-3-3 with GSK-3β, each Flag-tagged 14-3-3 isoform expression vector was transfected into CCE cells, immunoprecipitated with an anti-Flag antibody, then immunoblotted with anti-GSK-3β antibody. To investigate the phosphorylated residue of GSK-3β that is involved in interaction with 14-3-3σ, GSK-3β wild-type (WT), GSK-3β S9A mutant or GSK-3β T309A mutant constructs were co-transfected with 14-3-3σ-Flag vector. Transfected CCE cells were immunoprecipitated with anti-Flag antibody, then immunoblotted with anti-GSK-3β antibody.

For assay of β-catenin ubiquitination, CCE cells were co-transfected with 14-3-3σ-Flag or HA–β-catenin expression vectors for 46 h, then incubated with MG-132 (10 µM) for an additional 2 h. Cell lysates were harvested, immunoprecipitated with a mouse monoclonal anti-HA antibody, and immunoblotted with rabbit polyclonal antibodies against ubiquitin (Cell Signaling).

### GSK-3β Activity Assay

Assay of GSK-3β activity was based on measuring tau phosphorylation at Ser-396 and Ser-199 [36], [37] by using an ELISA kit (Invitrogen). In brief, CCE cells were co-transfected for 48 h with GSK-3β expression vector and 14-3-3σ-Flag vectors or their respective control vectors. The transfected CCE cells were lysed with RIPA buffer and immunoprecipitated with a specific antibody against GSK-3β. The immunoprecipitate was washed and incubated with an assay buffer containing 100 µM ATP and recombinant Tau proteins, then with an antibody against phospho-Ser396 of Tau, a secondary antibody, and substrates. The reaction was terminated by adding a stop reagent, and the optical density of the sample was analyzed at 450 nm in an ELISA reader. Values of Tau phosphor-Ser396 were normalized to the total protein level of Tau.

### Statistical Analysis

Differences between groups were analyzed by Student *t* test. A *p* value less than 0.05 was considered statistically significant.

## Results

### 14-3-3σ Promotes Mouse ES Cell (mESC) Proliferation

Retinoic acid (RA) is known to induce differentiation and suppress proliferation of mESC [38], [39]. However, the underlying mechanism is not entirely clear. To investigate whether 14-3-3 proteins are involved in regulating the actions of RA, we analyzed 14-3-3 isoforms in RA-treated mESCs. Among seven 14-3-3 isoforms, only 14-3-3σ was significantly reduced by RA (Figure 1A). To determine the role of 14-3-3σ in mESC proliferation, we treated CCE cells with two 14-3-3σ siRNA sequences. Using a previously reported sequence [28], [29], our results confirm that 14-3-3σ siRNA specifically suppressed 14-3-3σ proteins without changing the expression of other 14-3-3 isoforms (Figure 1B). Knockdown of 14-3-3σ resulted in reduced CCE viable cell numbers (Figure 1C) and BrdU incorporation (Figure 1D) at 48 h. The inhibitory effect of 14-3-3σ knockdown was confirmed by another siRNA sequence (Figure S1). We next evaluated the influence of 14-3-3 overexpression on CCE proliferation. CCE cells were transfected with each isoform of 14-3-3 and the isoform protein overexpression was verified by Western blotting (Figure 2A). Only 14-3-3σ overexpression increased CCE cell numbers (Figure 2B). Furthermore, overexpression of 14-3-3σ significantly increased viable cell numbers (Figure 2C, upper panel) and BrdU incorporation (Figure 2D) and partially rescued RA-induced decline in cell number (Figure 2C, lower panel). To ensure that the involvement of 14-3-3σ in mESC proliferation is not limited to CCE, we evaluated the effect of 14-3-3σ in other mESC proliferation. Overexpression of 14-3-3σ in R1 and D3 mESC significantly increased viable cell number and BrdU incorporation to an extent comparable to CCE cells (Figure S2). Taken together, these results indicate that 14-3-3σ plays a critical role in CCE proliferation.

### 14-3-3σ Promotes mESC Proliferation via β-catenin

To determine whether β-catenin is involved in 14-3-3σ-mediated mESC proliferation, CCE cells were co-transfected with 14-3-3σ overexpression vector and β-catenin siRNA. Suppression of β-catenin expression in CCE transfected with a previously described siRNA [30], [31] (Figure 3A) resulted in abrogation of 14-3-3σ-induced cell number increases (Figure 3B). These results were confirmed by another siRNA sequence of β-catenin which exhibited a lesser effect on suppressing β-catenin and a correlated lesser effect on reducing CCE proliferation (Figure S3). To investigate the relationship between 14-3-3σ and β-catenin, we transfected CCE cells with 14-3-3σ and analyzed β-catenin phosphorylation, ubiquitination, degradation and nuclear translocation. Phosphorylation and ubiquitination of β-catenin were significantly decreased (Figure 4A and 4B), whereas total β-catenin protein level was increased (Figure 4C, upper panel) in 14-3-3σ overexpressed CCE cells. Furthermore, β-catenin protein levels were increased in the nuclear fraction (Figure 4C, lower panel). Thus, 14-3-3σ prevents β-catenin from phosphorylation, ubiquitination and degradation, thereby increasing β-catenin protein stability and nuclear translocation.

To ensure that nuclear β-catenin is active in promoting β-catenin/Tcf-targeted gene expression in mESCs, we analyzed β-catenin transcriptional activity by using TOPFLASH/FOPFLASH reporters. 14-3-3σ overexpression increased the promoter activity by ∼5-fold over the control (Figure 4D). We next determined the expression of cyclin D1, one of the β-catenin target genes that are critical in cell cycle progression and cell proliferation. Cyclin D1 protein level was increased by ∼3-fold in 14-3-3σ-overexpressed cells (Figure 4E). Taken together, the results indicate that 14-3-3σ promotes mESC proliferation via β-catenin.

### 14-3-3σ Interacts with GSK-3β and Suppresses GSK-3β Activity

We performed immuneprecipitation (IP) to determine interaction of 14-3-3σ with GSK-3β as well as proteins in the β-catenin destruction complex. CCE cells were transfected with Flag-tagged 14-3-3σ, 14-3-3σ protein complex was pulled down by IP using a Flag antibody and interacting proteins were analyzed with Western blotting. 14-3-3σ was co-immunoprecipitated with GSK-3β but not β-catenin, axin or APC (Figure 5A. upper panel). 14-3-3σ was also co-immunoprecipitated with GSK-3β in R1 and D3 mESC (Figure 5A, middle and lower panels). To determine whether GSK-3β selectively interacts with 14-3-3σ isoform, we transfected CCE with each Flag-tagged 14-3-3 isoform, immunoprecipitated the complex with Flag antibody and analyzed GSK-3β protein by Western blotting. GSK-3β was co-precipitated only with 14-3-3σ (Figure 5B). These results suggest that 14-3-3σ binds GSK-3β in 14-3-3σ transfected CCE cells. Binding and catalytic activities of GSK-3β are regulated by phosphorylation. We prepared GSK-3β mutants for potential 14-3-3 binding sites at Ser9 (S9A) or Thr309 (T309A), constructed the mutant into the expression vector and analyzed the influence of mutant overexpression on interaction with 14-3-3σ. 14-3-3σ interaction with GSK-3β was reduced by S9A mutation but not T309A (Figure 5C). These results suggest that 14-3-3σ binding of GSK-3β requires phosphorylation at Ser9. Since PI-3K/Akt pathway is involved in GSK-3β Ser9 phosphorylation, we evaluated the effect of wortmannin, a PI-3K inhibitor on interaction of GSK-3β with 14-3-3σ. Wortmannin blocked the interaction in a concentration-dependent manner (Figure 5D).

We suspected that 14-3-3σ binding of GSK-3β might result in GSK-3β sequestration and inactivation. To address this, we analyzed the level of phosphorylated GSK-3β (pGSK-3β) and GSK-3β catalytic activity in 14-3-3σ overexpressed CCE cells. Compared to non-transfected cells, GSK-3β catalytic activity was significantly reduced in 14-3-3σ transfected cells (Figure 5E). In accordance with reduction of the catalytic activity, pGSK-3β was enhanced by 14-3-3σ overexpression which was abrogated by wortmannin induced Akt dephosphorylation and inactivation (Figure 5F). These results indicate that 14-3-3σ overexpression enhances GSK-3β phosphorylation and induces 14-3-3σ binding and sequestration of phosphorylated GSK-3β in a PI-3K/Akt dependent manner.

### 14-3-3σ Overexpression Disrupts Association of β-catenin with the Multiprotein Destruction Complex

Since 14-3-3σ overexpression induces GSK-3β binding and inactivation, we reasoned that 14-3-3σ overexpression could reduce β-catenin association with GSK-3β in the multiprotein destruction complex. To assess this, CCE cells were co-transfected with/without HA-tagged β-catenin and 14-3-3σ vectors. Association of β-catenin with APC, axin or GSK-3β was analyzed by immunoprecipitation followed by immunoblotting. Overexpression of 14-3-3σ significantly reduced association of β-catenin with GSK-3β as well as APC and axin, and increased the β-catenin level (Figure 6A). Besides, the binding of overexpression or endogenous β-catenin with p-GSK-3β is barely detected (Figure 6A). In addition, transfection of S9A GSK-3β mutant abolished 14-3-3σ-induced β-catenin expression while transfection of T309A did not (Figure 6B). These results are consistent with the interpretation that 14-3-3σ level is pivotal in controlling GSK-3β action and regulating β-catenin bioavailability. To provide additional evidence for this, we evaluated the effect of RA on pGSK-3β and β-catenin levels. RA treatment suppressed 14-3-3σ-increased pGSK-3β and reduced p-β-catenin (Figure 6C). 14-3-3σ transfection restored pGSK-3β and increased β-catenin reduced by RA (Figure 6C). Furthermore, RA treatment for 48 h slightly reduced Oct3/4 level. However, 14-3-3σ did not significantly affect Oct3/4 expression (Figure 6C). These results indicate that 14-3-3σ may regulate mouse ES cell proliferation without influence stemness and pluripotency. To further evaluate the endogenous association of 14-3-3σ with GSK-3β, we performed the IP experiments and found that GSK-3β interacts with endogenous 14-3-3σ (Figure 6D). Treatment of RA significantly reduced 14-3-3σ binding with GSK-3β (Figure 6D). Taken together, these results indicate high 14-3-3σ levels increase β-catenin by releasing β-catenin from GSK-3β and confer resistance to RA-induced β-catenin degradation by maintaining GSK-3β phosphorylation.

### 14-3-3σ Acts in Concert with Wnt to Control GSK-3β

As anticipated, Wnt3a increased pGSK-3β and β-catenin in CCE cells (Figure 7A). 14-3-3σ overexpression augmented Wnt3a-induced pGSK-3β phosphorylation and β-catenin elevation (Figure 7A). DKK1, an inhibitor of Wnt signaling blocked the action of Wnt3a on pGSK-3β and β-catenin but had a lesser effect on 14-3-3σ transfected CCE cells (Figure 7A). We next determined the GSK-3β binding by 14-3-3σ in the presence of Wnt3a and 14-3-3σ transfection. In the absence of 14-3-3σ overexpression, we detected lower interaction between endogenous 14-3-3σ and GSK-3β (Figure 7B). 14-3-3σ binding of GSK-3β was detected in 14-3-3σ transfected cells which were enhanced by Wnt3a treatment (Figure 7B). DKK1 did not have a significant effect on interaction of 14-3-3σ with GSK-3β in 14-3-3σ transfected cells treated with Wnt3a (Figure 7B). These results suggest high levels of 14-3-3σ augment the Wnt signaling to enhance GSK-3β inactivation and increase β-catenin stability.

## Discussion

Our findings provide important information regarding the novel role of 14-3-3σ in regulating mESC proliferation. Despite the expression of all seven isoforms of 14-3-3 proteins in mESCs, only 14-3-3σ participates in mESC proliferation by binding, sequestration and inactivating GSK-3β. Our results demonstrate that 14-3-3σ overexpression enhances GSK-3β phosphorylation and inactivation as well as increases interaction between 14-3-3σ and GSK-3β. Furthermore, 14-3-3σ overexpression triggers dissociation of β-catenin from the APC/axin/GSK-3β complex, the so-called multiprotein destruction complex. Since the transcriptional bioavailability of β-catenin is tightly controlled by GSK-3β in the destruction complex, our data lead us to conclude that 14-3-3σ is capable of sequestering GSK-3β and thereby releasing β-catenin from the multiprotein destruction complex which translocates into the nucleus and carries out the proliferative transcription.

GSK-3β inactivation depends on phosphorylation within the multiprotein destruction complex. At resting state, GSK-3β is active in phosphorylating β-catenin to induce its degradation via ubiquitination/proteasome. When stimulated by Wnt, GSK-3β is phosphorylated and dissociated from the multiprotein destruction complex, thus releasing β-catenin. It is generally thought that phosphorylated GSK-3β is rapidly dephosphorylated and reassociated with the APC/axin complex. In this study, we provide evidence that phosphorylated GSK-3β is controlled by 14-3-3σ. High levels of 14-3-3σ sequester and inactivate GSK-3β via which they enhance Wnt signaling to increase β-catenin. It is interesting that DKK blocks the effect of Wnt3a as expected but did not interfere with action of 14-3-3σ on GSK-3β binding. These findings indicate that 14-3-3σ provides a discrete pathway to control GSK-3β availability and activity.

It is well recognized that RA induces ES cells to undergo differentiation and proliferation arrest. A number of mechanisms of RA actions have been proposed but the exact mechanisms are not clear. We show in this study that 14-3-3σ/GSK-3β pathway is involved in RA-induced inhibition of mESC proliferation. RA selectively suppresses 14-3-3σ. It increases β-catenin phosphorylation and reduces β-catenin resulting in reduction of mESC proliferation. High levels of 14-3-3σ confer resistance to RA by restoring GSK-3β phosphorylation and sequestration. Thus, 14-3-3σ is pivotal in regulating GSK-3β/β-catenin bioavailability as illustrated in Fig. 8.

Our results reveal that knockdown of 14-3-3σ with siRNA reduces mESC proliferation by only 30–40% compared to control (Figure 1C and 1D), suggesting that mESC proliferation does not depend entirely on 14-3-3σ. A compensatory effect may be regulated by other signal pathways. This notion was supported by a recent report which indicates that 14-3-3σ-deleted mESC give rise to viable mice with B-cell developmental defects [40]. It is interesting that of all seven 14-3-3 isoforms expressed in mESCs, only 14-3-3σ is involved in regulating β-catenin-mediated mESC proliferation. In contrast, 14-3-3ζ was reported to bind GSK-3β and enhances Tau phosphorylation in brain [19], and 14-3-3ζ was reported to facilitate β-catenin export from the nucleus and thereby reduces β-catenin transcriptional activity [22]. Reasons for differential regulation of GSK-3β and β-catenin by different 14-3-3 isoforms in different tissues and cells are unclear and require further investigation.

According to the result in association of 14-3-3σ with GSK-3β S9A mutant, our results indicate that 14-3-3σ binds preferentially phosphorylated GSK-3β (Figure 5C). Using a pharmacological inhibitor of PI-3K, we show that PI-3K/Akt is required for 14-3-3σ binding of GSK-3β. GSK-3β phosphorylation at Ser9 and Thr309 are potential residues for 14-3-3 interaction. Mutation of GSK-3β Ser9 abrogates GSK-3β binding to 14-3-3σ whereas mutation of Thr309 did not alter its binding. These results suggest that PI-3K/Akt plays an essential role in promoting 14-3-3σ binding and sequestration of GSK-3β by Ser9 phosphorylation. PI-3K/Akt is recognized as an important signaling pathway for promoting ESC proliferation. We demonstrate that enhanced 14-3-3σ/GSK-3β interaction is an important downstream mechanism by which PI-3K/Akt mediates ESC proliferation and renewal.

It is interesting to note that β-catenin siRNA does not completely abrogate the enhancing action of 14-3-3σ on cell proliferation (Figure 3B). It is possible that 14-3-3σ may enhance cell proliferation by multiple mechanisms. Besides the Wnt/β-catenin mechanism, 14-3-3σ may bind phosphorylated Raf-1, activate Raf-1 and its downstream signaling pathway [41], [42]. Moreover, 14-3-3 was found to regulate the mammalian target of rapamycin (mTOR) pathway by interacting with tuberous sclerosis complex 2 (TSC2), and sequestering it from binding to mTOR complex, thereby increasing the mTOR activity on de novo protein synthesis and cell proliferation [43], [44].

In summary, this study shows for the first time that 14-3-3σ regulates mESC proliferation by binding and sequestering GSK-3β as well as inducing GSK-3β phosphorylation and inactivation in a PI-3K/Akt-dependent manner. 14-3-3σ is a novel target for ES cell expansion.

## Supporting Information

Figure S1 **14-3-3σ knockdown suppressed cell proliferation.** CCE cells were transfected with scramble or 14-3-3σ siRNA (Invitrogen, sence: GCGCAUCAUCGAU UCUGCCCGGUCA; antisence: UGACCGGGCAGAAUCGAUGAUGCGC) for 24 h or 48 h. (A) Knockdown of 14-3-3σ expression was determined by Western blot analysis after transfection for 48 h. (B) Proliferation and viable cell numbers of CCE cells were determined by trypan blue assay, and (C) BrdU assay. Each bar represents mean ± s.d. (n = 3). N.S. denotes statistically not significant; *, *p*<0.05.
(EPS)

Figure S2 **14-3-3σ transfection increased (A) cell numbers (B) BrdU incorporation in R1 and D3 mES cells.** Each bar is mean ± s.d. of three independent experiments. * *p*<0.05.
(EPS)

Figure S3 **β-catenin knockdown suppressed 14-3-3σ-enhanced cell proliferation.** CCE cells were co-transfected with control or 14-3-3σ overexpression vectors, and scramble or β-catenin siRNA (Invitrogen, sence: CCCAGAAUGCCGUUCGCCUUCAUUA; antiscene: UAAUGAAGGCGAACGGCAUUCUGGG) for 48 h. (A) Protein level of reduced β-catenin was confirmed by Western blot analysis after transfection for 48 h. (B) Proliferation of CCE cells was determined by trypan blue assay after 48 h transfection. Each bar represents mean ± s.d. (n = 3). *, *p*<0.05; **, *p*<0.01.
(EPS)

## References

[1] Evans MJ, Kaufman MH (1981). Establishment in culture of pluripotential cells from mouse embryos.. Nature.

[2] Martin GR (1981). Isolation of a pluripotent cell line from early mouse embryos cultured in medium conditioned by teratocarcinoma stem cells.. Proc Natl Acad Sci U S A.

[3] Bouhon IA, Joannides A, Kato H, Chandran S, Allen ND (2006). Embryonic stem cell-derived neural progenitors display temporal restriction to neural patterning.. Stem Cells.

[4] Sakurai H, Okawa Y, Inami Y, Nishio N, Isobe K (2008). Paraxial mesodermal progenitors derived from mouse embryonic stem cells contribute to muscle regeneration via differentiation into muscle satellite cells.. Stem Cells.

[5] Liu N, Lu M, Tian X, Han Z (2007). Molecular mechanisms involved in self-renewal and pluripotency of embryonic stem cells.. J Cell Physiol.

[6] Okita K, Yamanaka S (2006). Intracellular signaling pathways regulating pluripotency of embryonic stem cells.. Curr Stem Cell Res Ther.

[7] Katoh M (2007). WNT signaling pathway and stem cell signaling network.. Clin Cancer Res.

[8] Kleber M, Sommer L (2004). Wnt signaling and the regulation of stem cell function.. Curr Opin Cell Biol.

[9] Doble BW, Woodgett JR (2003). GSK-3: tricks of the trade for a multi-tasking kinase.. J Cell Sci.

[10] Nelson WJ, Nusse R (2004). Convergence of Wnt, beta-catenin, and cadherin pathways.. Science.

[11] Zeng X, Tamai K, Doble B, Li S, Huang H (2005). A dual-kinase mechanism for Wnt co-receptor phosphorylation and activation.. Nature.

[12] Aubert J, Dunstan H, Chambers I, Smith A (2002). Functional gene screening in embryonic stem cells implicates Wnt antagonism in neural differentiation.. Nat Biotechnol.

[13] Kielman MF, Rindapaa M, Gaspar C, van Poppel N, Breukel C (2002). Apc modulates embryonic stem-cell differentiation by controlling the dosage of beta-catenin signaling.. Nat Genet.

[14] Sato N, Meijer L, Skaltsounis L, Greengard P, Brivanlou AH (2004). Maintenance of pluripotency in human and mouse embryonic stem cells through activation of Wnt signaling by a pharmacological GSK-3-specific inhibitor.. Nat Med.

[15] Welsh GI, Wilson C, Proud CG (1996). GSK3: a SHAGGY frog story.. Trends Cell Biol.

[16] Fu H, Subramanian RR, Masters SC (2000). 14-3-3 proteins: structure, function, and regulation.. Annu Rev Pharmacol Toxicol.

[17] Morrison DK (2009). The 14-3-3 proteins: integrators of diverse signaling cues that impact cell fate and cancer development.. Trends Cell Biol.

[18] Yaffe MB, Rittinger K, Volinia S, Caron PR, Aitken A (1997). The structural basis for 14-3-3:phosphopeptide binding specificity.. Cell.

[19] Agarwal-Mawal A, Qureshi HY, Cafferty PW, Yuan Z, Han D (2003). 14-3-3 connects glycogen synthase kinase-3 beta to tau within a brain microtubule-associated tau phosphorylation complex.. J Biol Chem.

[20] Mwangi S, Anitha M, Fu H, Sitaraman SV, Srinivasan S (2006). Glial cell line-derived neurotrophic factor-mediated enteric neuronal survival involves glycogen synthase kinase-3beta phosphorylation and coupling with 14-3-3.. Neuroscience.

[21] Tian Q, Feetham MC, Tao WA, He XC, Li L (2004). Proteomic analysis identifies that 14-3-3zeta interacts with beta-catenin and facilitates its activation by Akt.. Proc Natl Acad Sci U S A.

[22] Li FQ, Mofunanya A, Harris K, Takemaru K (2008). Chibby cooperates with 14-3-3 to regulate beta-catenin subcellular distribution and signaling activity.. J Cell Biol.

[23] Chang TC, Chen YC, Yang MH, Chen CH, Hsing EW (2010). Rho kinases regulate the renewal and neural differentiation of embryonic stem cells in a cell plating density-dependent manner.. PLoS One.

[24] Liou JY, Ellent DP, Lee S, Goldsby J, Ko BS (2007). Cyclooxygenase-2-derived prostaglandin e2 protects mouse embryonic stem cells from apoptosis.. Stem Cells.

[25] Doetschman TC, Eistetter H, Katz M, Schmidt W, Kemler R (1985). The in vitro development of blastocyst-derived embryonic stem cell lines: formation of visceral yolk sac, blood islands and myocardium.. J Embryol Exp Morphol.

[26] Wood SA, Allen ND, Rossant J, Auerbach A, Nagy A (1993). Non-injection methods for the production of embryonic stem cell-embryo chimaeras.. Nature.

[27] Korinek V, Barker N, Morin PJ, van Wichen D, de Weger R (1997). Constitutive transcriptional activation by a beta-catenin-Tcf complex in APC−/− colon carcinoma.. Science.

[28] Wilker EW, Grant RA, Artim SC, Yaffe MB (2005). A structural basis for 14-3-3sigma functional specificity.. J Biol Chem.

[29] Yang HY, Wen YY, Chen CH, Lozano G, Lee MH (2003). 14-3-3 sigma positively regulates p53 and suppresses tumor growth.. Mol Cell Biol.

[30] Liu P, Yang J, Pei J, Pei D, Wilson MJ (2010). Regulation of MT1-MMP activity by beta-catenin in MDCK non-cancer and HT1080 cancer cells.. J Cell Physiol.

[31] Yan W, Tai HH (2006). Glycogen synthase kinase-3 phosphorylation, T-cell factor signaling activation, and cell morphology change following stimulation of thromboxane receptor alpha.. J Pharmacol Exp Ther.

[32] Zhou BP, Deng J, Xia W, Xu J, Li YM (2004). Dual regulation of Snail by GSK-3beta-mediated phosphorylation in control of epithelial-mesenchymal transition.. Nat Cell Biol.

[33] Ko BS, Chang TC, Shyue SK, Chen YC, Liou JY (2009). An efficient transfection method for mouse embryonic stem cells.. Gene Ther.

[34] Liou JY, Ko BS, Chang TC (2010). An efficient transfection method for mouse embryonic stem cells.. Methods Mol Biol.

[35] Liou JY, Ghelani D, Yeh S, Wu KK (2007). Nonsteroidal anti-inflammatory drugs induce colorectal cancer cell apoptosis by suppressing 14-3-3epsilon.. Cancer Res.

[36] Ding Q, He X, Xia W, Hsu JM, Chen CT (2007). Myeloid cell leukemia-1 inversely correlates with glycogen synthase kinase-3beta activity and associates with poor prognosis in human breast cancer.. Cancer Res.

[37] Ishiguro K, Shiratsuchi A, Sato S, Omori A, Arioka M (1993). Glycogen synthase kinase 3 beta is identical to tau protein kinase I generating several epitopes of paired helical filaments.. FEBS Lett.

[38] Duester G (2008). Retinoic acid synthesis and signaling during early organogenesis.. Cell.

[39] Martin-Ibanez R, Urban N, Sergent-Tanguy S, Pineda JR, Garrido-Clua N (2007). Interplay of leukemia inhibitory factor and retinoic acid on neural differentiation of mouse embryonic stem cells.. J Neurosci Res.

[40] Su YW, Hao Z, Hirao A, Yamamoto K, Lin WJ (2011). 14-3-3sigma regulates B-cell homeostasis through stabilization of FOXO1.. Proc Natl Acad Sci U S A.

[41] Fantl WJ, Muslin AJ, Kikuchi A, Martin JA, MacNicol AM (1994). Activation of Raf-1 by 14-3-3 proteins.. Nature.

[42] Li S, Janosch P, Tanji M, Rosenfeld GC, Waymire JC (1995). Regulation of Raf-1 kinase activity by the 14-3-3 family of proteins.. EMBO J.

[43] Li Y, Inoki K, Yeung R, Guan KL (2002). Regulation of TSC2 by 14-3-3 binding.. J Biol Chem.

[44] Shumway SD, Li Y, Xiong Y (2003). 14-3-3beta binds to and negatively regulates the tuberous sclerosis complex 2 (TSC2) tumor suppressor gene product, tuberin.. J Biol Chem.

